# Redetermination of (NH_4_)_2_HAsO_4_

**DOI:** 10.1107/S1600536812043565

**Published:** 2012-10-27

**Authors:** Matthias Weil

**Affiliations:** a Institute for Chemical Technologies and Analytics, Division of Structural Chemistry, Vienna University of Technology, Getreidemarkt 9/164-SC, A-1060 Vienna, Austria

## Abstract

In comparison with the original determination based on Weissenberg film data [Khan *et al.* (1970 ▶). *Acta Cryst.* B**26**, 1889–1892], the current redetermination of diammonium hydrogenarsenate(V) reveals all atoms with anisotropic displacement parameters and all H atoms localized. This allowed an unambiguous assignment of the hydrogen-bonding pattern, which is similar to that of the isotypic phosphate analogue (NH_4_)_2_HPO_4_. The structure of the title compound consists of slightly distorted AsO_3_(OH) and NH_4_ tetra­hedra, linked into a three-dimensional structure by an extensive network of O—H⋯O and N—H⋯O hydrogen bonds.

## Related literature

For the previous determination of (NH_4_)_2_HAsO_4_, see: Khan *et al.* (1970 ▶). The arsenate compound is isotypic with the phosphate analogue (NH_4_)_2_HPO_4_ (Khan *et al.*, 1972 ▶), for which another modification with *Z*′ = 2 has also recently been described (Kunz *et al.*, 2010 ▶).

## Experimental

### 

#### Crystal data

(NH_4_)_2_HAsO_4_
*M* *_r_* = 176.01
Monoclinic, 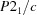

*a* = 11.3426 (4) Å
*b* = 6.8512 (3) Å
*c* = 8.1130 (3) Å
β = 113.784 (4)°
*V* = 576.92 (4) Å^3^
*Z* = 4
Mo *K*α radiation
μ = 5.82 mm^−1^
*T* = 293 K
0.14 × 0.12 × 0.02 mm

#### Data collection

Bruker APEXII CCD diffractometer
Absorption correction: multi-scan (*SADABS*; Bruker, 2009 ▶) *T*_min_ = 0.496, *T*_max_ = 0.873
6318 measured reflections
1674 independent reflections
1413 reflections with *I* > 2σ(*I*)
*R*_int_ = 0.033

#### Refinement

*R*[*F* ^2^ > 2σ(*F* ^2^)] = 0.022
*wR*(*F* ^2^) = 0.055
*S* = 1.04
1676 reflections
101 parameters
All H-atom parameters refined
Δρ_max_ = 0.59 e Å^−3^
Δρ_min_ = −0.84 e Å^−3^

### 

Data collection: *APEX2* (Bruker, 2009 ▶); cell refinement: *SAINT* (Bruker, 2009 ▶); data reduction: *SAINT*; program(s) used to solve structure: *SHELXS97* (Sheldrick, 2008 ▶); program(s) used to refine structure: *SHELXL97* (Sheldrick, 2008 ▶); molecular graphics: *ATOMS* (Dowty, 2006) ▶; software used to prepare material for publication: *publCIF* (Westrip, 2010 ▶).

## Supplementary Material

Crystal structure: contains datablock(s) I, global. DOI: 10.1107/S1600536812043565/hb6976sup1.cif

Structure factors: contains datablock(s) I. DOI: 10.1107/S1600536812043565/hb6976Isup2.hkl

Additional supplementary materials:  crystallographic information; 3D view; checkCIF report

## Figures and Tables

**Table 1:** Hydrogen-bond geometry (Å, °)

*D*—H⋯*A*	*D*—H	H⋯*A*	*D*⋯*A*	*D*—H⋯*A*
N1—H3*N*1⋯O2^i^	0.87 (3)	1.88 (3)	2.750 (2)	178 (3)
N1—H1*N*1⋯O3^ii^	0.91 (3)	1.91 (3)	2.780 (3)	158 (2)
N1—H2*N*1⋯O3^i^	0.89 (3)	2.06 (3)	2.930 (3)	167 (2)
N1—H4*N*1⋯O3^iii^	0.92 (3)	1.86 (3)	2.777 (2)	173 (3)
N2—H4*N*2⋯O2	0.92 (3)	2.00 (3)	2.910 (2)	174 (2)
N2—H2*N*2⋯O2^i^	0.89 (3)	1.93 (3)	2.809 (2)	174 (2)
N2—H1*N*2⋯O4	0.83 (3)	2.02 (3)	2.840 (2)	171 (3)
N2—H3*N*2⋯O4^iv^	0.85 (3)	1.95 (3)	2.793 (2)	176 (3)
O1—H1*O*⋯O4^v^	0.73 (3)	1.89 (3)	2.613 (2)	171 (4)

Symmetry codes: (i) 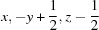; (ii) 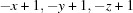; (iii) 
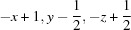
; (iv) 
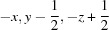
; (v) 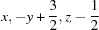.

**Fig. 1.**
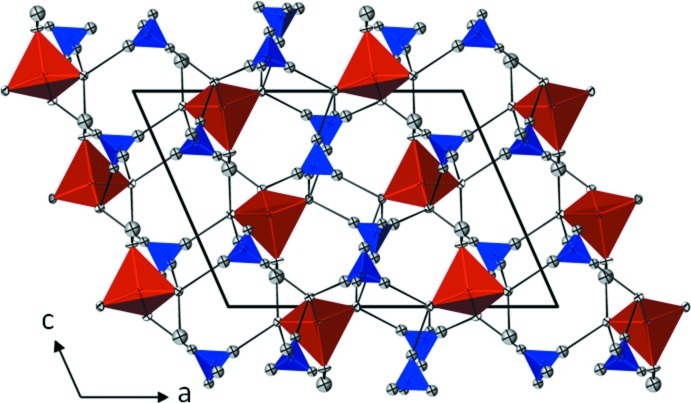
Projection of the crystal structure along [010]. AsO4 tetrahedra are red, NH4 tetrahedra are blue, O atoms are white, and H atoms are grey. Atoms are displayed with displacement ellipsoids at the 50% probability level. H···O hydrogen bonds are displayed with black lines.

## supplementary crystallographic information

### Comment

(NH_4_)_2_HAsO_4_ is a frequently used precursor material for preparation of arsenate(V) compounds, starting either from (aqueous) solutions or *via* ceramic routes. The crystal structure of (NH_4_)_2_HAsO_4_ has originally been determined by Khan *et al.* (1970) based on Weissenberg photographs. In the original study all atoms were refined with isotropic displacement parameters. Since H atoms could not be localized, the authors could make only assumptions with respect to the resulting hydrogen bonding pattern, deduced from N···O and O···O distances. These assumptions included three models: i) the NH_4_ ions exhibit rotatory oscillations; ii) the NH_4_ ions are in static disorder; iii) each of the two N atoms forms a bifurcated bond in addition to three normal hydrogen bonds (Khan *et al.*, 1970). Somewhat later Khan *et al.* (1972) showed for the isotypic phosphate analogue (NH_4_)_2_HPO_4_ that dynamic or static disorder can be ruled out for the NH_4_ groups and that the ammonium tetrahedra form four classical hydrogen bonds to the PO_3_(OH) groups. The current redetermination of the structure of (NH_4_)_2_HAsO_4_ using modern CCD-based data was intended to shed some light on its hydrogen bonding pattern and to compare the results with the phosphate analogue.

The redetermination confirmed the basic features of the original study, however with the unambiguous localization of all H atoms and, as expected, with higher precision and accuracy. Like in (NH_4_)_2_HPO_4_, the ammonium groups show no static or dynamic disorder and four normal N—H···O hydrogen bonds are formed between the constituents. The largest difference between the two determinations pertains to the O···O distance of the O—H···O (O1···O4) hydrogen bond. In the original study this distance was determined as 2.669 (13) Å, whereas it is 2.613 (2) Å in this study. The latter matches very well with 2.615 (1) Å for the phosphate analogue for which all H atoms could be localized (Khan *et al.*, 1972). In the latter study it was suggested that the difference between these O···O distances of the phosphate (2.615 (1) Å) and the arsenate (2.669 (13) Å) structure is a consequence of the different size of the P^5+^ and the As^5+^ ions. However, the current redetermination of (NH_4_)_2_HAsO_4_ shows that the influence of the different sizes for the phosphate (average P—O distance 1.54 Å) and the arsenate (average As—O distance 1.68 Å) tetrahedra can in fact be neglected.

Fig. 1 shows the structural set-up of the two different NH_4_ and the AsO_3_(OH) tetrahedra. All tetrahedra show slight angular distortions; the difference in As—O bond lengths for the three As—O bonds (average 1.674 Å) and the longer As—OH bond (1.7291 (15) Å) is normal. The ammonium cations and hydrogenarsenate anions are linked into a three-dimensional network by classical O—H···O and N—H···O hydrogen bonds, the numerical details of which are given in Table 1. The latter are very similar to those of the isotypic phosphate (NH_4_)_2_HPO_4_ (Khan *et al.*, 1972).

For (NH_4_)_2_HPO_4_ another crystalline polymorph has been described, resulting from hydrolysis of the educt, *viz.* ammonium hexafluoridophosphate (Kunz *et al.*, 2010). It would be interesting to know whether an arsenate polymorph isotypic with the phosphate analogue or another polymorph (NH_4_)_2_HAsO_4_ exist as well.

### Experimental

Crystals of the title compound were grown from an aqueous solution containing diluted arsenic acid (20%_wt_) mixed with a concentrated aqueous solution of ammonia in excess. The solution was kept in a desiccator with CaCl_2_ as drying agent. The first crystals, mostly with a plate-like form, appeared approximately after one week.

### Refinement

For better comparison, the same unit cell setting as in the previous determination of (NH_4_)_2_HAsO_4_ (Khan *et al.*, 1970) was used. The current setting is not reduced and can be transformed to the reduced setting by application of the matrix (101, 010, 001). For refinement, the atomic coordinates of the As, O and N atoms (Khan *et al.*, 1972) were used as starting parameters. All H atoms were clearly discernible from difference Fourier maps and were refined freely.

### Crystal data

**Table:**

(NH_4_)_2_HAsO_4_	*F*(000) = 352
*M**_r_* = 176.01	*D*_x_ = 2.026 Mg m^−^^3^
Monoclinic, *P*2_1_/*c*	Mo *K*α radiation, λ = 0.71073 Å
Hall symbol: -P 2ybc	Cell parameters from 2800 reflections
*a* = 11.3426 (4) Å	θ = 3.6–30.0°
*b* = 6.8512 (3) Å	µ = 5.82 mm^−^^1^
*c* = 8.1130 (3) Å	*T* = 293 K
β = 113.784 (4)°	Plate, colourless
*V* = 576.92 (4) Å^3^	0.14 × 0.12 × 0.02 mm
*Z* = 4	

### Data collection

**Table:**

Bruker APEXII CCD diffractometer	1674 independent reflections
Radiation source: fine-focus sealed tube	1413 reflections with *I* > 2σ(*I*)
Graphite monochromator	*R*_int_ = 0.033
ω– and φ–scans	θ_max_ = 30.0°, θ_min_ = 2.0°
Absorption correction: multi-scan (*SADABS*; Bruker, 2009)	*h* = −15→15
*T*_min_ = 0.496, *T*_max_ = 0.873	*k* = −6→9
6318 measured reflections	*l* = −11→10

### Refinement

**Table:**

Refinement on *F*^2^	Primary atom site location: structure-invariant direct methods
Least-squares matrix: full	Secondary atom site location: difference Fourier map
*R*[*F*^2^ > 2σ(*F*^2^)] = 0.022	Hydrogen site location: inferred from neighbouring sites
*wR*(*F*^2^) = 0.055	All H-atom parameters refined
*S* = 1.04	*w* = 1/[σ^2^(*F*_o_^2^) + (0.0304*P*)^2^] where *P* = (*F*_o_^2^ + 2*F*_c_^2^)/3
1676 reflections	(Δ/σ)_max_ = 0.001
101 parameters	Δρ_max_ = 0.59 e Å^−^^3^
0 restraints	Δρ_min_ = −0.84 e Å^−^^3^

### Special details

**Table:**

Geometry. All e.s.d.'s (except the e.s.d. in the dihedral angle between two l.s. planes) are estimated using the full covariance matrix. The cell e.s.d.'s are taken into account individually in the estimation of e.s.d.'s in distances, angles and torsion angles; correlations between e.s.d.'s in cell parameters are only used when they are defined by crystal symmetry. An approximate (isotropic) treatment of cell e.s.d.'s is used for estimating e.s.d.'s involving l.s. planes.
Refinement. Refinement of *F*^2^ against ALL reflections. The weighted *R*-factor *wR* and goodness of fit *S* are based on *F*^2^, conventional *R*-factors *R* are based on *F*, with *F* set to zero for negative *F*^2^. The threshold expression of *F*^2^ > σ(*F*^2^) is used only for calculating *R*-factors(gt) *etc*. and is not relevant to the choice of reflections for refinement. *R*-factors based on *F*^2^ are statistically about twice as large as those based on *F*, and *R*- factors based on ALL data will be even larger.

### Fractional atomic coordinates and isotropic or equivalent isotropic displacement parameters (Å^2^)

**Table:**

	*x*	*y*	*z*	*U*_iso_*/*U*_eq_	
As1	0.249593 (18)	0.89261 (3)	0.42786 (3)	0.00648 (7)	
O1	0.20879 (15)	0.9789 (2)	0.2115 (2)	0.0126 (3)	
O2	0.25894 (13)	0.0977 (2)	0.5442 (2)	0.0098 (3)	
O3	0.38858 (13)	0.7692 (2)	0.4982 (2)	0.0114 (3)	
O4	0.13017 (13)	0.7484 (2)	0.4289 (2)	0.0098 (3)	
N1	0.44933 (18)	0.1231 (3)	0.1532 (3)	0.0111 (3)	
N2	0.12140 (18)	0.3798 (3)	0.2643 (3)	0.0100 (3)	
H1N1	0.494 (3)	0.131 (3)	0.275 (4)	0.017 (7)*	
H2N1	0.422 (2)	0.003 (4)	0.114 (4)	0.013 (6)*	
H3N1	0.388 (3)	0.209 (4)	0.117 (4)	0.022 (7)*	
H4N1	0.509 (3)	0.167 (4)	0.111 (4)	0.016 (7)*	
H1N2	0.118 (3)	0.491 (5)	0.302 (4)	0.020 (7)*	
H2N2	0.169 (3)	0.392 (3)	0.201 (4)	0.013 (7)*	
H3N2	0.046 (3)	0.336 (4)	0.209 (4)	0.022 (7)*	
H4N2	0.164 (2)	0.297 (4)	0.358 (4)	0.013 (6)*	
H1O	0.186 (3)	0.907 (4)	0.139 (5)	0.032 (10)*	

### Atomic displacement parameters (Å^2^)

**Table:**

	*U*^11^	*U*^22^	*U*^33^	*U*^12^	*U*^13^	*U*^23^
As1	0.00865 (10)	0.00455 (11)	0.00669 (11)	0.00021 (7)	0.00355 (7)	−0.00027 (8)
O1	0.0228 (7)	0.0084 (8)	0.0071 (7)	−0.0016 (6)	0.0064 (6)	−0.0004 (6)
O2	0.0134 (7)	0.0062 (7)	0.0105 (7)	−0.0002 (5)	0.0056 (6)	−0.0026 (6)
O3	0.0102 (6)	0.0113 (7)	0.0131 (7)	0.0031 (5)	0.0051 (6)	0.0011 (6)
O4	0.0111 (6)	0.0068 (7)	0.0119 (7)	−0.0018 (5)	0.0051 (5)	0.0003 (6)
N1	0.0117 (8)	0.0096 (9)	0.0123 (9)	0.0008 (7)	0.0052 (7)	0.0006 (7)
N2	0.0121 (8)	0.0071 (9)	0.0120 (9)	0.0003 (7)	0.0059 (7)	−0.0005 (7)

### Geometric parameters (Å, º)

**Table:**

As1—O2^i^	1.6718 (14)	N1—H2N1	0.89 (3)
As1—O3	1.6732 (14)	N1—H3N1	0.87 (3)
As1—O4	1.6793 (14)	N1—H4N1	0.92 (3)
As1—O1	1.7293 (15)	N2—H1N2	0.83 (3)
O1—H1O	0.73 (3)	N2—H2N2	0.89 (3)
O2—As1^ii^	1.6718 (14)	N2—H3N2	0.85 (3)
N1—H1N1	0.91 (3)	N2—H4N2	0.92 (3)
			
O2^i^—As1—O3	113.29 (7)	H1N1—N1—H4N1	103 (2)
O2^i^—As1—O4	111.04 (7)	H2N1—N1—H4N1	112 (2)
O3—As1—O4	110.62 (7)	H3N1—N1—H4N1	105 (2)
O2^i^—As1—O1	102.46 (7)	H1N2—N2—H2N2	105 (2)
O3—As1—O1	110.40 (7)	H1N2—N2—H3N2	110 (3)
O4—As1—O1	108.67 (7)	H2N2—N2—H3N2	116 (3)
As1—O1—H1O	117 (3)	H1N2—N2—H4N2	111 (3)
H1N1—N1—H2N1	114 (2)	H2N2—N2—H4N2	107 (2)
H1N1—N1—H3N1	110 (2)	H3N2—N2—H4N2	108 (2)
H2N1—N1—H3N1	113 (3)		

Symmetry codes: (i) *x*, *y*+1, *z*; (ii) *x*, *y*−1, *z*.

### Hydrogen-bond geometry (Å, º)

**Table:**

*D*—H···*A*	*D*—H	H···*A*	*D*···*A*	*D*—H···*A*
N1—H3*N*1···O2^iii^	0.87 (3)	1.88 (3)	2.750 (2)	178 (3)
N1—H1*N*1···O3^iv^	0.91 (3)	1.91 (3)	2.780 (3)	158 (2)
N1—H2*N*1···O3^iii^	0.89 (3)	2.06 (3)	2.930 (3)	167 (2)
N1—H4*N*1···O3^v^	0.92 (3)	1.86 (3)	2.777 (2)	173 (3)
N2—H4*N*2···O2	0.92 (3)	2.00 (3)	2.910 (2)	174 (2)
N2—H2*N*2···O2^iii^	0.89 (3)	1.93 (3)	2.809 (2)	174 (2)
N2—H1*N*2···O4	0.83 (3)	2.02 (3)	2.840 (2)	171 (3)
N2—H3*N*2···O4^vi^	0.85 (3)	1.95 (3)	2.793 (2)	176 (3)
O1—H1*O*···O4^vii^	0.73 (3)	1.89 (3)	2.613 (2)	171 (4)

Symmetry codes: (iii) *x*, −*y*+1/2, *z*−1/2; (iv) −*x*+1, −*y*+1, −*z*+1; (v) −*x*+1, *y*−1/2, −*z*+1/2; (vi) −*x*, *y*−1/2, −*z*+1/2; (vii) *x*, −*y*+3/2, *z*−1/2.
